# Assessing food security and environmental quality under policy stringency and geopolitics using counterfactual and machine learning approaches

**DOI:** 10.1038/s41598-026-48181-3

**Published:** 2026-04-17

**Authors:** Md. Idris Ali, Mohammad Bin Amin, Mohammad Salahuddin, Md. Nahin Hossain, Judit Oláh

**Affiliations:** 1 Department of Business and Technology Management, Islamic University of Technology (IUT), Board Bazar, Gazipur, Bangladesh; 2 Toronto Metropolitan University, Toronto, Canada; 3 Doctoral School of Management and Business, Faculty of Economics and Business, University of Debrecen, Böszörményi street 138, Debrecen, 4032 Hungary; 4 Department of Business Administration, Faculty of Business Studies, Army University of Science and Technology, Nilphamari, Saidpur, 5310 Bangladesh; 5 North South University, Bashundhara R/A, Dhaka, 1229 Bangladesh; 6 Department of Business and Technology Management, Islamic University of Technology (IUT), Board Bazar, Gazipur, Bangladesh; 7 Doctoral School of Management and Business Administration, John von Neumann University, Kecskemét, 6000 Hungary; 8 Faculty of Economics and Business, University of Debrecen, Böszörményi street 138, Debrecen, 4032 Hungary; 9 Department of Trade and Finance, Faculty of Economics and Management, Czech University of Life Sciences Prague, Prague, Czech Republic; 10 Department of Business Studies, State University of Bangladesh, 696 Kendua, Kanchan, Rupganj, Narayanganj, 1461, Dhaka, Bangladesh

**Keywords:** Food security, Policy stringency, Geopolitics, Carbon emissions, Ecological footprint, Ecology, Ecology, Environmental sciences, Environmental social sciences

## Abstract

The provision of food security promotes sustainable economic growth by fostering healthier and more productive populations. However, achieving food security can impose environmental costs, as production and distribution processes contribute to deforestation, greenhouse gas emissions, and resource depletion. Environmental policy stringency plays a critical role in mitigating these impacts by regulating industrial practices and promoting sustainable technologies. This study examines the relationship between food security and environmental policy stringency in shaping greenhouse gas emissions and ecological footprints, while accounting for energy consumption, geopolitical risks, and technological innovation. Using Canadian annual time series data from 1990 to 2022, the study employs the dynamic autoregressive distributed lag (DARDL) model to analyze long-run dynamics. The empirical results indicate that a 1% increase in food security raises CO₂ emissions by 0.16% and ecological footprint by 0.14%, confirming its environmentally detrimental effect. Energy consumption exerts the largest impact, increasing CO₂ emissions by 0.60% and ecological footprint by 0.67%. Geopolitical risk contributes positively to environmental degradation, increasing CO₂ emissions by 0.01% and ecological footprint by 0.79%. In contrast, environmental policy stringency reduces CO₂ emissions by 0.13% and ecological footprint by 0.16%, while technological innovation decreases emissions by 0.11% and ecological footprint by 0.10% in the long run. All estimated coefficients are statistically significant at conventional levels. Counterfactual analysis further evaluates the effects of ± 1% and ± 5% shocks among variables, revealing asymmetric environmental responses. The robustness of the findings is confirmed using Kernel-based Regularized Least Squares (KRLS). These results suggest that policymakers must balance food security objectives with environmental sustainability by strengthening environmental regulations and promoting green agricultural technologies.

##  Introduction

Food security is a fundamental pillar of sustainable economic development, as it ensures adequate nutrition, enhances human productivity, and supports long-term societal welfare^1^. However, achieving food security presents significant environmental challenges. The expansion of agricultural production, food processing, and distribution systems intensifies energy consumption, accelerates resource depletion, and contributes substantially to greenhouse gas (GHG) emissions and ecological degradation^2^. With the global population projected to reach nearly 10 billion by 2050, food production must increase by approximately 50% compared to 2010 levels, thereby placing unprecedented pressure on environmental systems and natural resources.

The environmental consequences of food security are closely linked to energy use and production practices. Modern agricultural systems rely heavily on fossil fuel–based inputs, including mechanization, fertilizers, and transportation networks, which significantly increase carbon emissions and ecological footprints^3^. A growing body of empirical literature confirms that fossil fuel consumption remains a key driver of environmental degradation, while eco-friendly technologies can mitigate these adverse effects^4^. Similarly, evidence highlights that non-renewable energy consumption significantly exacerbates CO₂ emissions and environmental pressure^5^. Empirical evidence further indicates that the global food system is a major contributor to environmental degradation, accounting for nearly one-quarter of total GHG emissions^1,6–8^. These dynamics highlight the urgent need to reconcile food security objectives with environmental sustainability through effective policy interventions.

Environmental policy stringency has emerged as a key regulatory instrument for addressing environmental degradation. Policies such as carbon pricing, emissions trading systems, renewable energy mandates, and strict industrial standards are designed to reduce emissions and promote sustainable resource use^9^. Recent evidence suggests that adaptive and well-designed policy interventions can generate co-benefits across environmental, agricultural, and economic systems, improving both ecological outcomes and productivity^10^. In parallel, technological innovation—particularly in clean energy systems and sustainable agricultural practices—plays a crucial role in improving environmental efficiency^11^. Green innovation and environmentally oriented fiscal policies have been shown to significantly enhance renewable energy adoption and environmental sustainability^12^. Furthermore, technological advancement, financial development, and foreign direct investment (FDI) influence environmental outcomes through complex channels, potentially mitigating or exacerbating environmental degradation depending on institutional and technological conditions^13^. Despite these policy and technological advancements, global emissions and ecological footprints continue to rise due to population growth, industrialization, and unsustainable consumption patterns^14^. This raises important questions regarding the effectiveness of environmental policies and their interaction with food security objectives.

In addition to policy and technological factors, geopolitical risks have become an increasingly important determinant of environmental outcomes. Geopolitical tensions influence environmental quality through several transmission mechanisms. First, supply chain disruptions resulting from conflicts or trade restrictions can lead to production inefficiencies and increased emissions. Second, volatility in global energy markets may force countries to shift toward more carbon-intensive energy sources, thereby exacerbating environmental degradation. Third, trade barriers and political instability may compel nations to rely on less efficient domestic production systems, increasing pressure on local ecosystems^15–17^. In the context of agricultural systems, such disruptions may also affect procurement efficiency and production organization, further amplifying environmental and economic inefficiencies^18^. These mechanisms underscore the complex and indirect ways in which geopolitical risks shape the relationship between food security and environmental sustainability.

Recent studies also emphasize the importance of resilience in food systems, particularly under climate change and external shocks. Strengthening food security resilience through adaptive strategies and institutional support is critical for maintaining stable food systems while minimizing environmental costs^19^. Additionally, governance structures—such as fiscal decentralization and public expenditure allocation—play an important role in achieving sustainable development outcomes by influencing environmental policy effectiveness and resource distribution^20^. These insights highlight the need for integrated frameworks that consider economic, environmental, and institutional dimensions simultaneously.

Despite a growing body of literature on food security and environmental quality, several important gaps remain. First, existing studies often examine food security and environmental policy in isolation, with limited attention to their joint effects on environmental outcomes. Second, while prior research has explored the roles of energy consumption, technological innovation, financial development, and institutional factors^4,13,20^, the role of geopolitical risks within this integrated framework remains underexplored. Third, there is a scarcity of studies employing advanced econometric approaches, such as the dynamic autoregressive distributed lag (DARDL) model, to capture long-run dynamics and counterfactual relationships among these variables. Addressing these gaps is essential for developing a comprehensive understanding of the trade-offs between food security and environmental sustainability.

Canada is selected as the empirical case due to its distinctive economic and policy characteristics. As a high-income OECD country, Canada combines strong food security systems with stringent environmental regulations and high levels of energy consumption. The country is also highly integrated into global energy and food markets, making it particularly sensitive to geopolitical risks and trade disruptions. Moreover, Canada’s commitment to technological innovation and sustainable development makes it an appropriate case for analyzing the effectiveness of environmental policies in mitigating environmental degradation. These features provide valuable insights that are relevant for other advanced economies with similar institutional and policy frameworks.

Using annual time series data for Canada from 1990 to 2022, this study employs the dynamic autoregressive distributed lag (DARDL) model to examine long-run relationships and counterfactual dynamics among the variables. The findings indicate that food security, energy consumption, and geopolitical risks contribute to increased carbon emissions and ecological footprint, while environmental policy stringency and technological innovation promote environmental sustainability in the long run. The robustness of these results is further validated using Kernel-based Regularized Least Squares (KRLS), which captures potential nonlinear relationships.

This study makes several important contributions. First, it addresses a critical research gap by jointly analyzing food security, environmental policy stringency, and geopolitical risks within a single empirical framework. Second, it advances methodological rigor by applying the DARDL approach alongside machine learning techniques. Third, it provides policy-relevant insights for balancing food security objectives with environmental sustainability in the presence of global uncertainties. Finally, this study contributes to the literature by examining the interconnected effects of food security, environmental policy stringency, energy consumption, geopolitical risks, and technological innovation on environmental quality, proxied by carbon emissions and ecological footprint. By integrating these dimensions within a unified empirical framework, the study provides a more comprehensive analysis of environmental sustainability.

The remainder of the paper is structured as follows. Section 2 reviews the relevant literature. Section 3 outlines the data and methodology. Section 4 presents the empirical results. Section 5 provides the discussion. Finally, Sect. 5 concludes with policy implications and recommendations.

##  Literature review

This section comprises two essential and interrelated components: the theoretical framework and the review of empirical literature.

### Theoretical framework

The nexus between food security, environmental policy stringency, and environmental quality—measured through carbon emissions and ecological footprint—can be explained through multiple complementary theoretical perspectives. First, the Porter Hypothesis posits that stringent environmental regulations, although imposing short-term compliance costs, can stimulate innovation that enhances both environmental performance and economic competitiveness^21,22^. In the context of food systems, this implies that well-designed environmental policies can incentivize the adoption of cleaner agricultural technologies and energy-efficient production processes, thereby improving food security while reducing environmental degradation^23^. Second, the Energy–Environment Nexus highlights the central role of energy consumption—particularly fossil fuel dependence—in driving environmental degradation. Empirical studies demonstrate that non-renewable energy consumption significantly increases carbon emissions, whereas eco-friendly technologies can mitigate environmental impacts^4,5^. This framework is particularly relevant for food systems, which are highly energy-intensive across production, processing, and distribution stages. Third, the Sustainable Development and Institutional Theory emphasizes the role of governance, policy design, and institutional quality in achieving environmental sustainability. Recent evidence suggests that adaptive environmental policies and efficient governance structures can generate co-benefits across ecological, agricultural, and economic systems^10,20^. Moreover, financial development and FDI, when aligned with technological progress, can either mitigate or exacerbate environmental degradation depending on regulatory frameworks^13^. Finally, the concept of Resilient Food Systems underscores the importance of adaptability and robustness in maintaining food security under environmental and geopolitical shocks. Climate change, geopolitical instability, and market disruptions necessitate resilient food systems that balance productivity with sustainability^19^.

**Fig. 1:**
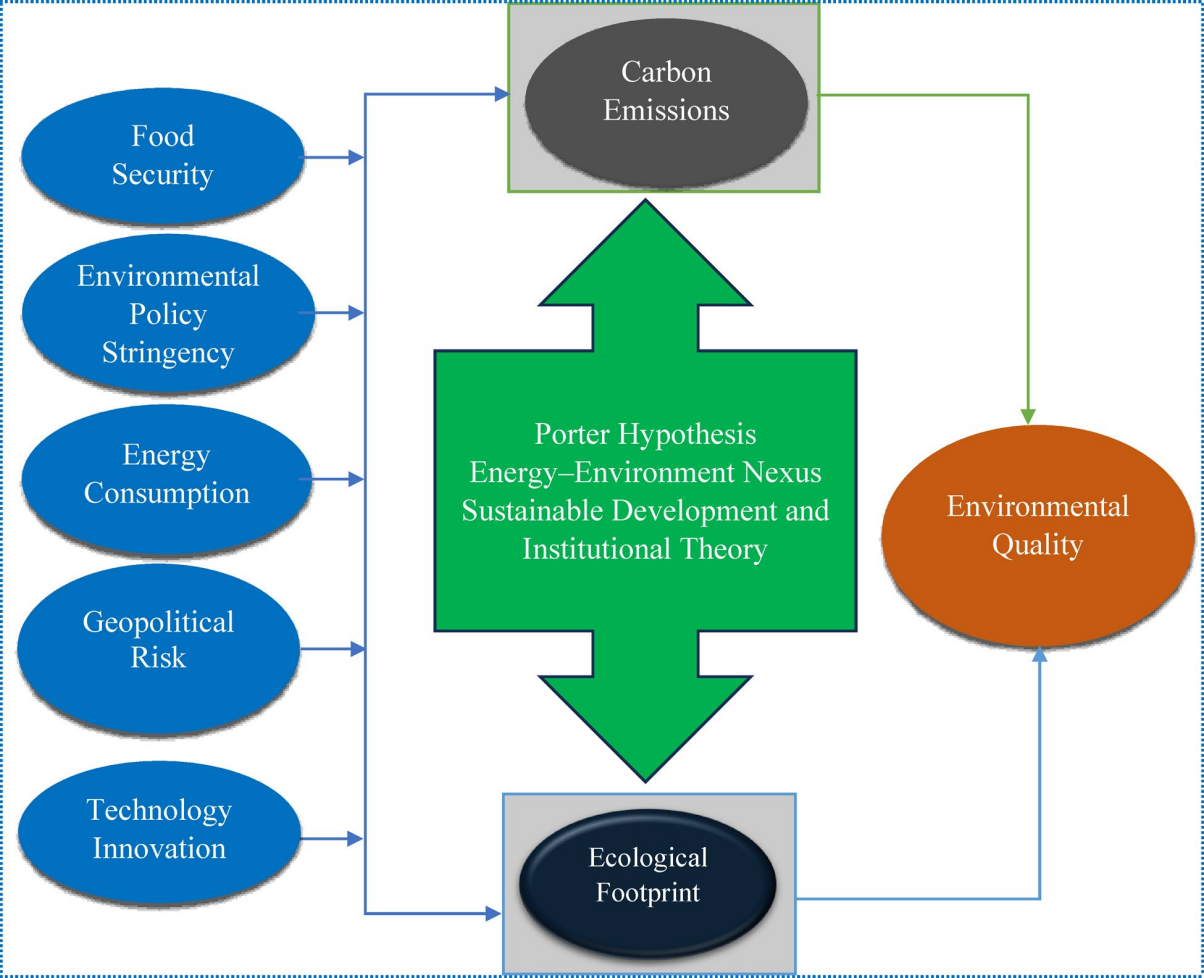
Conceptual framework.

Together, these theoretical perspectives provide a comprehensive framework for understanding how food security, environmental policy stringency, energy consumption, geopolitical risks, and technological innovation interact to shape environmental outcomes (Fig. 1).

### Empirical literature review

Food security is a critical component of sustainable development, closely linked to environmental outcomes such as carbon emissions and ecological footprint. Ensuring food security requires substantial resource inputs, including energy, water, and land, which often intensify environmental pressures. Agriculture, a core component of food systems, contributes significantly to GHG emissions through activities such as livestock production, deforestation, and energy-intensive cultivation practices^7,24^.

#### Food security, energy and environment

The concept of food security encompasses availability, access, utilization, and stability. Achieving these dimensions requires extensive use of natural and energy resources, which contributes to environmental degradation. The food system accounts for approximately one-quarter of global GHG emissions, highlighting its substantial environmental footprint^7^. Energy consumption plays a crucial role in this nexus. Fossil fuel-based agricultural practices—such as mechanization, irrigation, and fertilizer production—significantly increase carbon emissions and ecological footprints^25^. Empirical evidence confirms that non-renewable energy consumption is a major driver of environmental degradation, whereas the adoption of eco-friendly technologies can reduce emissions^4,5^. In addition, the structure and efficiency of agricultural systems influence environmental outcomes. For example, inefficient procurement and production systems can increase resource use and emissions, while optimized agricultural supply chains can enhance both economic and environmental efficiency^18^. Sustainable agricultural practices—such as agroecology and organic farming—have been shown to reduce environmental harm and improve long-term resilience, although their large-scale impacts remain underexplored^26^.

####  Policy stringency, technology and environment

Environmental policy stringency is a critical determinant of environmental performance. Stringent policies—such as carbon pricing, emissions trading systems, and renewable energy mandates—encourage shifts toward cleaner production and consumption patterns (OECD^27^^28^,;. The Porter Hypothesis suggests that such policies can stimulate technological innovation, leading to improved environmental outcomes^22^. Empirical studies support this view, showing that environmental regulations promote the adoption of green technologies and reduce emissions^29,30^.

Recent literature further highlights the role of green innovation and fiscal instruments in promoting sustainability. For instance, Aydin and Bozatli^12^ demonstrate that environmental taxes and financial development significantly enhance renewable energy consumption in OECD countries. Similarly, adaptive policy frameworks can generate co-benefits across environmental and economic dimensions, improving both productivity and sustainability^10^. Technological innovation and financial development also play a dual role. While advancements in clean technologies reduce emissions, poorly regulated financial flows or FDI may increase environmental degradation^13^. Therefore, the effectiveness of environmental policies depends on complementary institutional and technological factors. Despite these benefits, challenges such as policy resistance, carbon leakage, and institutional constraints can limit the effectiveness of environmental regulations^31,32^.

#### Geopolitics and environment

Geopolitical factors significantly influence environmental sustainability by shaping energy markets, trade patterns, and resource allocation. Countries with abundant fossil fuel resources often prioritize economic growth over environmental sustainability, resulting in higher emissions^33^. Geopolitical tensions can disrupt global energy markets, delay transitions to renewable energy, and increase reliance on carbon-intensive energy sources^34^. Additionally, trade restrictions and conflicts can lead to inefficient resource use and increased environmental degradation. Geopolitics also affects international cooperation on environmental issues. Rivalries among nations can hinder the effectiveness of global agreements such as the Paris Accord, limiting collective efforts to reduce emissions^35^. Furthermore, geopolitical instability often redirects investments away from sustainable technologies toward short-term priorities, reducing environmental progress^36^. These dynamics highlight the indirect but significant role of geopolitical risks in shaping environmental outcomes, particularly within globalized food and energy systems.

####  Research gap

Despite extensive research on food security, environmental policy, and environmental quality, several critical gaps remain. First, existing studies predominantly examine these factors in isolation, with limited attention to their integrated and interactive effects on environmental outcomes. Second, while recent studies have explored the roles of energy consumption, technological innovation, financial development, and institutional quality^4,13,20^, the role of geopolitical risks within this combined framework remains insufficiently addressed. Third, there is a notable lack of studies incorporating food system efficiency, resilience, and adaptive policy mechanisms into environmental analysis^10,18,19^. These dimensions are critical for understanding how food security interacts with environmental sustainability under real-world constraints. Fourth, from a methodological perspective, the literature is dominated by conventional econometric techniques such as ARDL, FMOLS, and PMG, while advanced approaches like the Dynamic Autoregressive Distributed Lag (DARDL) model and machine learning techniques such as Kernel-based Regularized Least Squares (KRLS) remain underutilized.

Therefore, this study addresses these gaps by providing an integrated analysis of food security, environmental policy stringency, energy consumption, geopolitical risks, and technological innovation, using advanced econometric and machine learning techniques to capture both long-run relationships and complex nonlinear dynamics.

## Research methodology

### Models

This study examines the impact of food security, environmental policy stringency, energy consumption, geopolitical risks, and technological innovation on environmental quality in Canada. The model specification is grounded in established theoretical frameworks, including the Porter Hypothesis, the energy–environment nexus, and sustainable development theory, which emphasize the roles of policy, energy use, and innovation in shaping environmental outcomes.

Existing empirical literature provides strong support for the inclusion of these variables. For instance, energy consumption has been widely identified as a key driver of carbon emissions and ecological degradation^4,5^. Similarly, environmental policy stringency is shown to reduce emissions by promoting cleaner technologies and improving resource efficiency^10,12^. Technological innovation plays a critical role in enhancing environmental sustainability by facilitating green production processes^11,13^. Moreover, recent studies highlight the importance of geopolitical risks in influencing environmental outcomes through energy market disruptions and trade uncertainties^15,16^. In addition, food security has been increasingly linked to environmental degradation due to its dependence on resource-intensive production systems^7,24^.

Based on these theoretical and empirical considerations, two models are specified to capture the effects of these variables on environmental quality using two distinct indicators: carbon emissions and ecological footprint.

Model 1: Food security – carbon emissions nexus

1 $$\ln CO_{2} = \alpha + \beta _{1} \ln FS_{t} + \beta _{2} EPS_{t} + \beta _{3} EC_{t} + \beta _{4} \ln GPR_{t} + \beta _{5} \ln TI_{t} + \varepsilon _{t}$$

Model 2: Food security – ecological footprint nexus

2 $$\ln EF = \alpha + \beta _{1} \ln FS_{t} + \beta _{2} EPS_{t} + \beta _{3} EC_{t} + \beta _{4} \ln GPR_{t} + \beta _{5} \ln TI_{t} + \varepsilon _{t}$$

Where, *lnCO*_*2*_ indicates per capita carbon emissions; *lnEF* denotes ecological footprint; *lnFS* expresses food security; *lnEPS* represents environmental policy stringency; *lnEC* expresses energy consumption; *lnGPR* depicts geopolitical risks; *lnTI* is technology innovation; *α* represents intercept; *β* indicates the coefficient of the variable; $\:\epsilon\:$ is the error term; and *t* represents time.

### Data details

Canada has been selected as a country of study to examine the impact of food security, environmental policy stringency, energy consumption, geopolitical risks, and technological innovation on carbon emissions and ecological footprint because it combines a vast resource base with progressive environmental policies. As a G7 nation, it offers valuable insights into the intersection of global climate diplomacy and domestic policy implementation. Furthermore, Canada’s advancements in clean technology and its reliance on energy-intensive industries provide a unique opportunity to analyze how innovation and economic structures influence environmental outcomes. All variables used in this study were obtained from internationally recognized databases. The dataset was carefully inspected for missing observations. In cases where minor gaps existed, linear interpolation was applied to ensure continuity of the time series. However, the dataset is largely complete, and no substantial data imputation was required. This ensures that the estimation results are not biased by missing data issues.

**Table 1:** Variable details.

Variables	Description	Sources
lnco_2_	Carbon dioxide emissions	World Bank^37^,
lnEF	Ecological footprint	York University Ecological Footprint Initiative & Global Footprint Network^38^
lnFS	Food security	World Bank^37^,
lnEPS	Environmental policy stringency	OECD^39^
lnEC	Total energy consumption	US Energy Information Administration (EIA^40^,
lnGPR	Global geopolitical risk index	Caldara and Iacoviello^41^,
lnTI	Technology innovation	World Bank^37^,

Data details are illustrated in Table 1, where carbon emissions (CO_2_) and ecological footprint serve as dependent variables, while the remaining variables act as independent variables. CO_2_ emissions data, sourced from the World Bank^37^, represent annual total emissions per capita. Ecological footprint data, obtained from the York University Ecological Footprint Initiative and Global Footprint Network^38^, measure the biologically productive land and sea area required to produce the resources a population consumes and absorb the waste it generates. This metric is a critical tool for evaluating the sustainability of resource use within the context of prevailing technology and management practices. Food security data, proxied by the Food Production Index, is also sourced from the World Bank^37^. The Food Production Index tracks changes in the production of food commodities relative to a base period by weighting each crop’s production by its economic importance and aggregating the results, excluding non-food items such as coffee and tobacco. Environmental Policy Stringency (EPS) data measure the strictness of environmental policies by assessing the explicit or implicit costs imposed on polluters. This index, scored on a scale from 0 (least stringent) to 6 (most stringent), includes policies such as carbon taxes, emissions trading systems, and renewable energy mandates, reflecting the intensity of regulations and their economic implications. Total energy consumption data, sourced from the US Energy Information Administration (EIA), represent the sum of all energy used by a country. This includes energy from fossil fuels, renewable sources, nuclear power, and electricity imports. Measured in British thermal units (BTUs), it provides a comprehensive view of energy demand across sectors such as industry, transportation, and residential use. The Geopolitical Risk Index, constructed by Caldara and Iacoviello^41^, quantifies the frequency of keywords related to geopolitical tensions—such as war, terrorism, and political instability—in prominent international newspapers. Using text-analysis techniques, the index identifies and counts these terms across predefined articles. It is standardized to ensure consistent measurement over time, enabling comparative analysis across different periods and geopolitical events. Finally, technology innovation data are proxied by the number of patent applications, sourced from the World Bank^37^. These data involve counting patent filings through the Patent Cooperation Treaty procedure or patent offices by residents of a country. Patent applications provide insight into the level of innovation and technological development, focusing specifically on filings by residents rather than non-residents.

### Statistical approaches

This section outlines the statistical methodologies employed, including a range of diagnostic tests performed before and after estimation, as well as the regression techniques utilized for data analysis.

#### Data stationarity test

This study employs the Phillips-Perron (PP) and Augmented Dickey-Fuller (ADF) tests to assess the stationarity of the variables, a crucial prerequisite for reliable econometric modeling. These tests detect the presence of a unit root, indicating whether a time series is non-stationary and influenced by stochastic trends. The ADF test builds on the original Dickey-Fuller method by including lagged differences of the dependent variable to address autocorrelation, while the PP test corrects for serial correlation and heteroskedasticity in the error terms without requiring lagged differences. By determining the order of integration—whether variables are stationary at levels [I(0)] or become stationary after differencing [I(1)]—these tests ensure the robustness of the analysis and help identify the appropriate econometric techniques, such as co-integration and counterfactual analysis, used in the study.

#### Bounds testing of autoregressive distributed lag (ARDL)

This study utilizes the bounds testing procedure to evaluate the co-integration of variables within the model, determining the existence of long-term relationships by analyzing the significance of the F-statistic at a 5% threshold. The method involves comparing the calculated F-statistic with the critical values established by Pesaran et al.^42^,. If the F-statistic surpasses the upper critical bound, the null hypothesis of no co-integration is rejected, indicating the presence of a long-term relationship. Conversely, if the F-statistic is below the lower bound, the null hypothesis is accepted, signifying no co-integration. The hypotheses for bounds testing are mathematically represented as:

$\:{H}_{0}={\sigma\:}_{1}={\sigma\:}_{2}={\sigma\:}_{3}=\dots\:={\sigma\:}_{n}=0$ (No co-integration).

$\:{H}_{1}={\sigma\:}_{1}\ne\:{\sigma\:}_{2}\ne\:{\sigma\:}_{3}\ne\:\dots\:\ne\:{\sigma\:}_{n}\ne\:0$ (Co-integration exists).

To examine these relationships, the ARDL bounds testing approach is applied using the following equation:

3 $$\begin{aligned} \Delta Y_{t} = & \vartheta _{0} + \vartheta _{1} Y_{{t - i}} + \vartheta _{2} X_{{1t - i}} + \vartheta _{3} X_{{2t - i}} + \vartheta _{4} X_{{3t - i}} + \ldots + \vartheta _{n} X_{{nt - i}} + \\ & + \mathop \sum \limits_{{i = 1}}^{r} \theta _{1} Y_{{t - i}} + \mathop \sum \limits_{{i = 1}}^{r} \theta _{2} X_{{1t - i}} + \mathop \sum \limits_{{i = 1}}^{r} \theta _{3} X_{{2t - i}} + \mathop \sum \limits_{{i = 1}}^{r} \theta _{4} X_{{3t - i}} + \ldots + \mathop \sum \limits_{{i = 1}}^{r} \theta _{n} X_{{nt - i}} + \varepsilon _{t} \\ \end{aligned}$$

Here, Δ denotes the first difference in the series, $\:t-i$ indicates to the lag length calculated by the Akaike Information Criterion (AIC), and $\:\vartheta\:$ and $\:\theta\:$ are coefficients capturing the long-term and short-term dynamics, respectively. $\:{\epsilon\:}_{t}$ represents the error term. After confirming a co-integrating relationship using the bounds test, the ARDL framework is employed for its robustness with small samples and mixed integration orders. The ARDL model for long-term relationships is expressed as:

4 $$Y_{t} = \varphi _{0} + \mathop \sum \limits_{{i = 1}}^{h} \delta _{1} Y_{{t - i}} + \mathop \sum \limits_{{i = 1}}^{h} \delta _{2} X_{{1t - i}} + \mathop \sum \limits_{{i = 1}}^{h} \delta _{3} X_{{2t - i}} + \mathop \sum \limits_{{i = 1}}^{h} \delta _{4} X_{{3t - i}} + \ldots + \mathop \sum \limits_{{i = 1}}^{h} \delta _{n} X_{{nt - i}} + \varepsilon _{t}$$

Here, $\:\delta\:$ represents the long-term coefficients, and the optimal lag structure is selected using the AIC. Short-term dynamics and the error correction mechanism are represented as:

5 $$Y_{t} = \varphi _{0} + \mathop \sum \limits_{{i = 1}}^{h} \partial _{1} Y_{{t - i}} + \mathop \sum \limits_{{i = 1}}^{h} \partial _{2} X_{{1t - i}} + \mathop \sum \limits_{{i = 1}}^{h} \partial _{3} X_{{2t - i}} + \mathop \sum \limits_{{i = 1}}^{h} \partial _{4} X_{{3t - i}} + \ldots + \mathop \sum \limits_{{i = 1}}^{h} \partial _{n} X_{{nt - i}} + \gamma ECT_{{t - 1}} + \varepsilon _{t}$$

Where, $\:\partial\:$ represents the short-term coefficients, and the Error Correction Term (ECT) denotes the rate of adjustment from short-term deviations to long-term equilibrium.

#### Simulation technique for DARDL

This study applies the Dynamic Autoregressive Distributed Lag (DARDL) framework introduced by Jordan and Philips^43^, an innovative econometric method designed to explore both short-term and long-term relationships in datasets with co-integration. The DARDL approach has gained increasing attention in environmental and energy economics due to its ability to capture dynamic adjustments and counterfactual responses. Recent studies have applied this framework to examine environmental sustainability and energy–emissions relationships, demonstrating its effectiveness in modeling asymmetric and dynamic interactions among variables^44,45^. The DARDL model is particularly well-suited for handling variables with mixed integration orders, specifically I(0) and I(1), making it highly flexible for analyzing complex data structures. This approach leverages simulation techniques to model the impacts of sudden changes in independent variables, providing a detailed analysis of how these shocks influence the dependent variable over time. Through these simulations, the model captures both direct and indirect effects, offering a robust understanding of variable interactions and their stability under diverse conditions.

To address situations where traditional bounds testing may be inadequate due to violations of assumptions, this study considers an enhanced ARDL bounds testing framework incorporating surface regression, as recommended by Kripfganz and Schneider^46^. The Error Correction Term (ECT) in the DARDL approach is represented as follows:

6 $$\begin{aligned} \Delta Y_{t} & = \varphi _{0} + \partial _{0} Y_{{t - 1}} + \beta _{1} X_{{1t}} + \partial _{1} X_{{1t - 1}} + \beta _{2} X_{{2t}} + \partial _{2} X_{{2t - 1}} + \beta _{3} X_{{3t}} \\ & + \partial _{3} X_{{3t - 1}} + \ldots + \beta _{n} X_{{nt}} + \partial _{n} X_{{nt - 1}} + \gamma ECT_{{t - i}} + \varepsilon _{t} \\ \end{aligned}$$

This study also employs the simulation approach outlined by Khan et al. (2019), performing 5,000 simulations based on a multivariate normal distribution. These simulations enable the estimation of the error correction mechanism, facilitating a comprehensive understanding of how variables return to equilibrium following shocks and enhancing the robustness of the analysis.

#### Machine learning approach from KRLS

For enhanced robustness, this study employs the Kernel Regularized Least Squares (KRLS) method, as outlined by Hainmueller and Hazlett^47^ and applied in Sarkodie and Owusu^48^. KRLS leverages radial basis function kernels to flexibly model non-linear relationships while maintaining interpretability akin to traditional regression models. A key feature of KRLS is its ability to estimate partial derivatives, providing detailed insights into the marginal effects of covariates. To ensure model reliability and prevent overfitting, KRLS incorporates a regularization term that balances model complexity with fit quality.

The Gaussian kernel, a foundational component of the KRLS methodology, is mathematically defined as:

7 $$k\left( {x_{j} ,x_{i} } \right) = e^{{ - \frac{{\left\| {x_{j} - x_{i}^{2} } \right\|}}{{\sigma ^{2} }}}}$$

where $\left\| {x_{j} - x_{i} ^{2} } \right\|$denotes the squared Euclidean distance between data points *x*_*i*_ and *x*_*j*_​, and $\:{\sigma\:}^{2}\:$is the kernel width parameter controlling the smoothness of the function. The kernel value is maximized when *x*_*i*_ = *x*_*j*_​, gradually decreasing with increasing distance between the points, ultimately approaching zero.

The predicted value at any specific point x^*^ is computed as a weighted sum of the kernel values:

8 $$\:y=f\left({x}^{*}\right)=\sum\:_{i=1}^{N}{c}_{i}k({x}^{*},{x}_{i})$$

where $\:{c}_{i}$​ depicts the weight assigned to each kernel value, capturing the contribution of each observation to the overall prediction.

The KRLS model represents the relationship between dependent and independent variables in matrix form:

9 $$y = Kc = \left[ {\begin{array}{*{20}c} {\begin{array}{*{20}c} {k\left( {x_{1} ,~x_{1} } \right)} & {k\left( {x_{1} ,~x_{2} } \right)} & { \ldots ~~~~k\left( {x_{1} ,~x_{N} } \right)} \\ {k\left( {x_{2} ,~x_{1} } \right)} & \ddots & ~ \\ \vdots & ~ & ~ \\ \end{array} } \\ {\begin{array}{*{20}c} {k\left( {x_{N} ,~x_{1} } \right)} & {~~~~~~~~~~~~~~~~~} & {~~~~~~~~k\left( {x_{N} ,~x_{N} } \right)} \\ \end{array} } \\ \end{array} } \right]\left[ {\begin{array}{*{20}c} {c_{1} } \\ {c_{2} } \\ {c_{N} } \\ \end{array} } \right]$$

The KRLS methodology incorporates a penalty term to ensure balance between model complexity and accuracy. This is expressed through the following objective function:

10 $$\:\begin{array}{c}argmin\\\:f\in\:H\end{array}\sum\:_{i}\left(V\left(f\left({x}_{i}\right),\:{y}_{i}\right)\right)+\lambda\:\mathcal{R}\left(f\right)$$

where *V[f*(*x*_*i*_), *y*_*i*_] measures the loss or discrepancy between the predicted values $\:f\left({x}_{i}\right)$ and actual outcomes $\:{y}_{i}$ and $\:\lambda\:$ (a positive regularization parameter) controls the trade-off between model complexity and fitting accuracy.

The KRLS algorithm applies Tikhonov regularization to optimize the objective function, resulting in the following minimization equation:

11 $$\:{c}^{*}=\:\begin{array}{c}argmin\\\:c\in\:{\mathbb{R}}^{D}\end{array}(y-Kc{)}^{T}(y-Kc)+\lambda\:{c}^{T}Kc$$

A notable feature of KRLS is its ability to compute pointwise partial derivatives, which allow exploration of marginal effects of input variables. The marginal effect of the *j*-th variable is given by:

By calculating pointwise partial derivatives, the influence of input variables on explanatory variables can be clarified in terms of their marginal effects, expressed as:

12 $$\frac{{\widehat{{\partial y}}}}{{dx_{j}^{{\left( d \right)}} }} = \frac{{ - 2}}{{\sigma ^{2} }}\mathop \sum \limits_{i} c_{i} e^{{\frac{{\left\| { - x_{i} - x_{j} ^{2} } \right\|}}{{\sigma ^{2} }}}} \left( {x_{i}^{{\left( d \right)}} - x_{j}^{{\left( d \right)}} } \right)$$

where $\:{x}_{j}^{\left(d\right)}$ indicates the *d*-th dimension of the input variable *x*_*j*_​, and the summation captures the influence of all observations weighted by their kernel similarity.

**Fig. 2:**
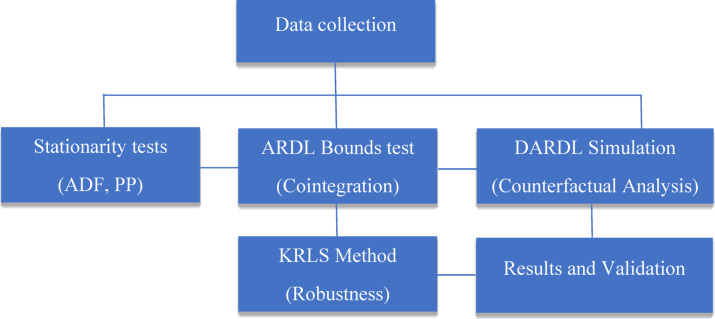
Summary of research methods.

To ensure the robustness of the KRLS results, sensitivity analysis was conducted by varying the kernel bandwidth parameter ($\:{\sigma\:}^{2}$) and regularization parameter ($\:\lambda\:$). The findings remained consistent across alternative parameter specifications, indicating that the estimated relationships are stable and not driven by model tuning. This enhances the reliability of the non-linear estimates obtained from the KRLS approach (Fig 2, ^47^).

## Findings and discussions

###  Descriptive statistics analysis

Table 2 provides the summary statistics for the study’s variables, offering insights into their central tendency, variability, and distribution. The dependent variable, *lnCO2*, exhibits minimal variation with a low standard deviation of 0.044, indicating stability during the study period. The independent variables—*lnEF* (ecological footprint), *lnFS* (food security), *lnEPS* (environmental policy stringency), *lnEC* (energy consumption), *lnGPR* (geopolitical risk), and *lnTI* (technology innovation)—demonstrate moderate variability, with their mean and standard deviation values reflecting consistency and balance across the dataset. The Jarque-Bera test results show insignificant p-values for all variables, confirming alignment with the assumption of normal distribution. Overall, the dataset appears suitable for further econometric analysis, including regression modeling and hypothesis testing.

**Table 2:** Summary statistics.

	lnco_2_	lnef	lnfs	lneps	lnec	lngpr	lnti
Mean	2.766	2.129	4.401	1.928	2.910	0.211	8.308
Median	2.760	2.119	4.399	2.056	2.907	0.180	8.340
Maximum	2.855	2.252	4.725	3.611	3.183	0.461	8.616
Minimum	2.689	2.047	4.105	0.500	2.577	0.096	7.843
Std. Dev.	0.044	0.051	0.187	1.127	0.150	0.087	0.198
Skewness	0.270	0.636	0.190	0.045	−0.202	1.306	−0.689
Kurtosis	2.304	2.865	1.938	1.385	2.986	4.029	2.882
Jarque-Bera	1.003	2.111	1.643	3.381	0.211	1.177	2.471
Probability	0.606	0.348	0.440	0.184	0.900	0.673	0.291
Observations	33	33	33	33	33	33	33

Source: Authors’ calculation.

**Fig. 3:**
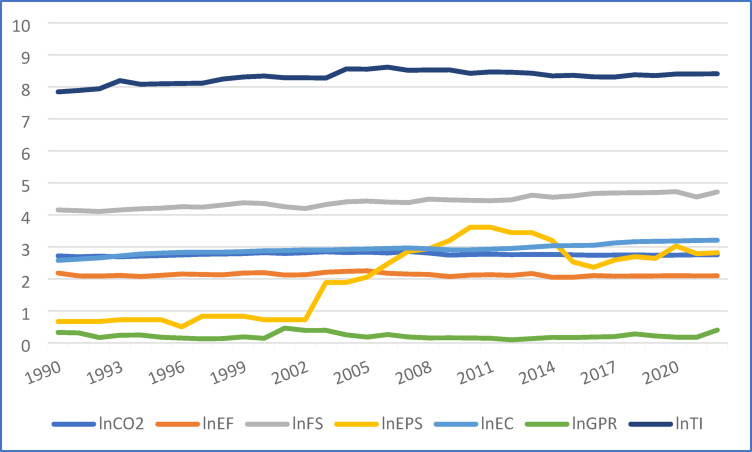
Trends in log-transformed environmental, energy, and economic indicators (1990–2022).

Figure 3 illustrates the time-series trends of the study variables from 1990 to 2022. The figure shows relatively stable movements in carbon emissions and ecological footprint, while environmental policy stringency exhibits noticeable fluctuations over time. Food security and technological innovation display gradual upward trends, whereas geopolitical risks remain comparatively volatile.

**Table 3:** Correlation matrix.

Variables	lnco_2_	lnef	lnfs	lneps	lnec	lngpr	lnti
lnco_2_	1.000						
lnef	0.653***	1.000					
lnfs	0.783***	−0.235*	1.000				
lneps	−0.660***	−0.379**	0.076	1.000			
lnec	0.724***	0.921***	−0.243	0.085	1.000		
lngpr	0.627***	0.689***	0.279	0.343*	0.716***	1.000	
lnti	−0.602***	0.202*	0.580***	0.206	0.350**	−0.198	1.000

Note: ***, ** and * indicate 1%, 5% and 10% level of significance, respectively.

Table 3 shows the correlation matrix, where *lnCO*_*2*_ is positively associated with food security, energy consumption, and geopolitical risk, indicating that increased levels of these factors contribute to higher emissions. These findings align with expectations, as resource-intensive activities linked to food security and energy demand often result in greater emissions, while geopolitical instability can disrupt environmental regulations. Conversely, *lnCO*_*2*_ is negatively correlated with environmental policy stringency and technology innovation, suggesting that stricter policies and advancements in technology are effective in reducing emissions by promoting sustainable practices and innovation. For *lnEF*, positive correlations are observed with energy consumption and geopolitical risk, emphasizing the impact of energy-intensive activities and geopolitical factors on the ecological footprint. There is also a moderate positive correlation with technology innovation, hinting at a potential relationship between innovation and ecological resource demands. In contrast, ecological footprint is negatively correlated with environmental policy stringency, highlighting the role of stringent policies in reducing ecological degradation. The relationship between ecological footprint and food security is less pronounced, suggesting variability in their connection depending on specific contexts.

**Table 4:** Unit root analysis.

Variables	Level (ADF)	1 st difference (ADF)	Level (PP)	1 st difference (PP)
lnco_2_	−1.536	−7.158***	−1.671	−6.926***
lnef	−3.029	−7.125***	−2.925	−6.526***
lnfs	−4.068***	−6.739***	−4.178***	−6.166***
lneps	−1.621	−4.343***	−1.303	−4.310***
lnec	−2.378	−6.155***	−2.203	−5.750***
lngpr	−2.740	−6.205***	−2.731	−6.162***
lnti	−2.018	−6.855***	−2.115	−6.697***

Note: Asterisk *** denotes 1% level of significance.

The unit root analysis, presented in Table 4, confirms the stationarity properties of the study variables using both the ADF and PP tests. The results indicate that none of the variables are stationary at levels [*I*(0)] except for *lnFS*, which is stationary at the 1% significance level. However, all variables become stationary after first differencing [*I*(1)] at a 1% level of significance. These results validate the use of the Dynamic Autoregressive Distributed Lag (DARDL) approach, which is specifically suited for datasets with mixed orders of integration .

**Table 5:** PSS bounds test results.

Models		K	Decision
Model 1: lnCO_2_=*f*(lnFS, lnEPS, lnEC, lnGPR, lnTI)	F	8.704***	Cointegrated
	t	−5.796***	
Model 2: lnEF = *f*(lnFS, lnCEP, lnEC, lnGPR, lnTI)	F	9.853***	Cointegrated
	t	−6.716***	

PSS critical values						
	10%		5%		1%	
	Level	1 st diff.	Level	1 st diff.	Level	1 st diff.
F	2.62	3.79	2.96	4.18	3.41	4.68
t	−2.86	−4.19	−3.13	−4.46	−3.43	−4.79

Note: Significance levels of 1% is represented by ***.

Table 5 depicts the results of the PSS (Pesaran, Shin, and Smith) bounds test, calculating the co-integration relationships among variables in two models. In Model 1, the *F*-statistic of 8.704 and the *t*-statistic of −5.796 both exceed the critical values at the 1% significance level, confirming the presence of co-integration. Similarly, in Model 2, the *F*-statistic of 9.853 and the *t*-statistic of −6.716 also surpass the 1% critical bounds, providing strong evidence of co-integration. These results indicate long-term relationships between the dependent variables and their respective explanatory variables in both models. The findings validate the appropriateness of the specified models for further analysis.

###  Key findings

Table 6 presents the DARDL estimation results, examining the effects of Canada’s food security and environmental policy stringency on two key environmental indicators: carbon emissions and ecological footprint. The Error Correction Term (ECT) coefficients for both Model 1 and Model 2 are highly significant at the 1% level, indicating a rapid adjustment to deviations from long-term equilibrium. Notably, carbon emissions and ecological footprint converge toward their equilibrium values at an impressive rate of 99% following disruptions driven by changes in food security. These results highlight the robustness and reliability of the correction mechanism, reinforcing the stability of the long-term relationships between the variables over time.

**Table 6:** Results of DARDL estimation.

Variables	Model 1: Food security–emissions nexus	Model 2: Food security–ecological footprint nexus
Error correction	−0.813*** (0.091)	−0.765*** (0.079)
Long run equation		
lnFS_t−1_	0.159*** (0.018)	0.136*** (0.214)
lnEPS_t−1_	−0.130** (0.053)	−0.158** (0.063)
lnEC_t−1_	0.599*** (0.193)	0.670*** (0.219)
lnGPR_t−1_	0.011** (0.004)	0.786*** (0.070)
lnTI_t−1_	−0.114** (0.048)	−0.103* (0.055)
Short run equation		
∆lnFS	−1.590*** (0.220)	0.864*** (0.282)
∆lnEPS	0.128 (0.152)	0.418** (0.179)
∆lnEC	0.018 (0.051)	0.074 (0.174)
∆lnGPR	−0.337** (0.133)	0.037 (0.030)
∆lnTI	0.013 (0.018)	−0.037 (0.412)
Constant	4.387***(0.929)	3.177*** (0.779)
R-sq.	0.851	0.896
Adjusted R-sq.	0.817	0.871
Total counts	33	33
Simulations	5000	5000

Note: ***, ** and * illustrate the 1%, 5% and 10% significance levels, respectively. The standard errors are in parentheses.

The DARDL estimation results for Model 1 and Model 2, as presented in Table 6, reveal a significant long-run positive association between food security (*lnFS*) and both carbon emissions (*lnCO*_*2*_) and ecological footprint (*lnEF*) at the 1% significance level. These findings suggest that, within the Canadian context, improvements in food security are linked to higher levels of carbon emissions and a greater ecological footprint. The findings further unveil that a 1% increase in environmental policy stringency (*lnEPS*) leads to improvements in environmental quality, evidenced by reductions in *lnCO*_*2*_ and *lnEF* in the long run by 0.13% and 0.16%, respectively. These negative relationships between *lnEPS* and the environmental indicators suggest that stringent environmental policies are effective tools for mitigating carbon emissions and ecological footprints in the Canadian context. Our findings also unpack a positive relationship between energy consumption (*lnEC*) and both *lnCO*_*2*_ and *lnEF*. Specifically, a 1% increase in *lnEC* results in a 0.60% increase in lnCO_2_ and a 0.67% increase in *lnEF* (Table 6). This increase in carbon emissions and ecological footprint due to energy consumption in the Canadian context implies that the country’s energy demand heavily relies on carbon-intensive sources, such as fossil fuels, which contribute significantly to environmental degradation. The results further reveal that a 1% increase in geopolitical risks (*lnGPR*) leads to a 0.01% increase in *lnCO*_*2*_ and a 0.79% increase in *lnEF*. This relationship suggests that heightened geopolitical risks contribute to higher carbon emissions and ecological footprints in the Canadian context. Finally, technology innovation (*lnTI*) contributes to improved environmental quality by reducing both carbon emissions and the ecological footprint. Specifically, a 1% increase in *lnTI* leads to a long-term decrease in *lnCO*_*2*_ and *lnEF* by 0.11% and 0.10%, respectively. This negative association between *lnTI* and environmental indicators suggests that advancements in technology play a critical role in mitigating environmental degradation in the Canadian context. Additionally, the results in Table 6 indicate a significant short-run relationship between *lnFS* and *lnGPR* with lnCO_2_, and a positive association is observed between *lnFS* and *lnEPS* with *lnEF*.

### Results from diagnostics tests

The diagnostic tests summarized in Table 6 confirm the appropriateness of the model by addressing concerns related to normality, heteroscedasticity, autocorrelation, and structural breaks. Furthermore, the adjusted R-squared values demonstrate that the independent variables explain 81.7% of the variation in the dependent variable for Model 1 and 87.1% for Model 2, indicating strong model performance.

**Table 7:** Autocorrelation test.

Lags(*p*)	Model 1			Model 2		
	F-statistic	Df	*p* > F	F-statistic	Df	*p* > F
1	0.561	(1, 24)	0.461	1.135	(1, 24)	0.297
2	0.434	(2, 23)	0.652	0.793	(2, 23)	0.465
3	0.311	(3, 22)	0.817	0.609	(3, 22)	0.616
4	0.308	(4, 21)	0.870	1.740	(4, 21)	0.179

At the 5% significance level, the Breusch-Godfrey LM (Lagrange Multiplier) test with four lags rejects the null hypothesis that no serial correlation exists (Table 7). This finding confirms the absence of autocorrelation in the residuals of the ARDL model, ensuring the reliability of the estimation.

**Table 8:** Cameron & Trivedi IM-test.

Statistic	Model 1			Model 2		
	$\:{\chi\:}^{2}$	Df	*p*-value	$\:{\chi\:}^{2}$	Df	*p*-value
Heteroskedasticity	28.04	20	0.110	20.72	20	0.413
Skewness	6.22	5	0.285	7.51	5	0.186
Kurtosis	1.79	1	0.181	0.00	1	0.819
Total	36.06	26	0.110	28.28	26	0.345

The findings from the Cameron and Trivedi (2005) IM test confirm the null hypothesis of homoscedasticity, demonstrating that the residuals maintain a uniform variance across two models, as shown in Table 8.

**Table 9:** Skewness/Kurtosis tests for normality.

Variable	Obs	Prob (Skewness)	Prob (Kurtosis)	Joint adj.$\:{\chi\:}^{2}$ (2)	Joint Prob> $\:{\chi\:}^{2}$
lnco_2_	33	0.364	0.637	1.11	0.574
lnef	33	0.301	0.477	1.69	0.430
lnfs	33	0.251	0.881	1.43	0.489
lneps	33	0.446	0.875	0.63	0.730
lnec	33	0.771	0.923	0.09	0.954
lngpr	33	0.381	0.824	3.37	0.113
lnti	33	0.609	0.553	4.36	0.113

As shown in Table 9, Skewness/Kurtosis tests (chi-square: $\:{\chi\:}^{2}$) validate the normality of residuals at the 5% significance level, indicating that residuals follow a normal distribution.

**Fig. 4:**
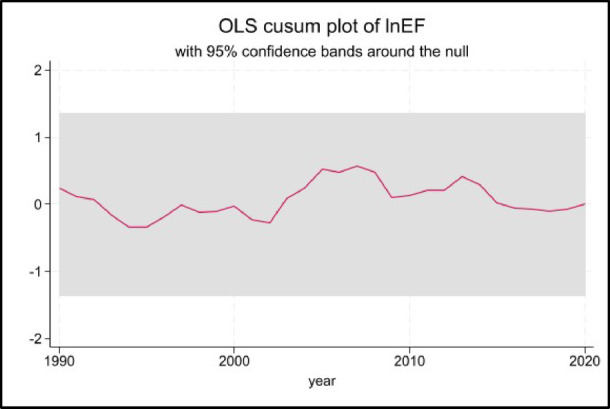
OLS CUSUM plot depicting the cumulative sum test for assessing parameter stability concerning Model 1.

**Fig. 5:**
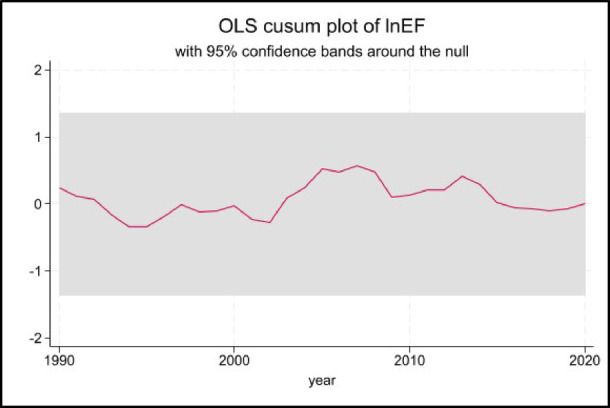
OLS CUSUM plot depicting the cumulative sum test for assessing parameter stability concerning Model 2.

The study utilizes cumulative sum (CUSUM) tests to assess the temporal stability of the model coefficients and mitigate the influence of potential structural breaks. Figures 4 and 5 illustrate that the test statistics for Models 1 and 2 remain consistently within the 95% confidence interval, providing strong evidence for the stability of the estimated parameters throughout the analysis period.

### Simulation graphs of DARDL approach

**Fig. 6:**
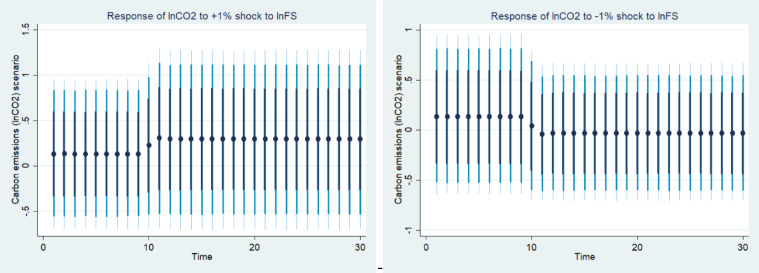
1% (±) shock to food security on carbon emissions yielded using the DARDL simulations area plot.

**Fig. 7:**
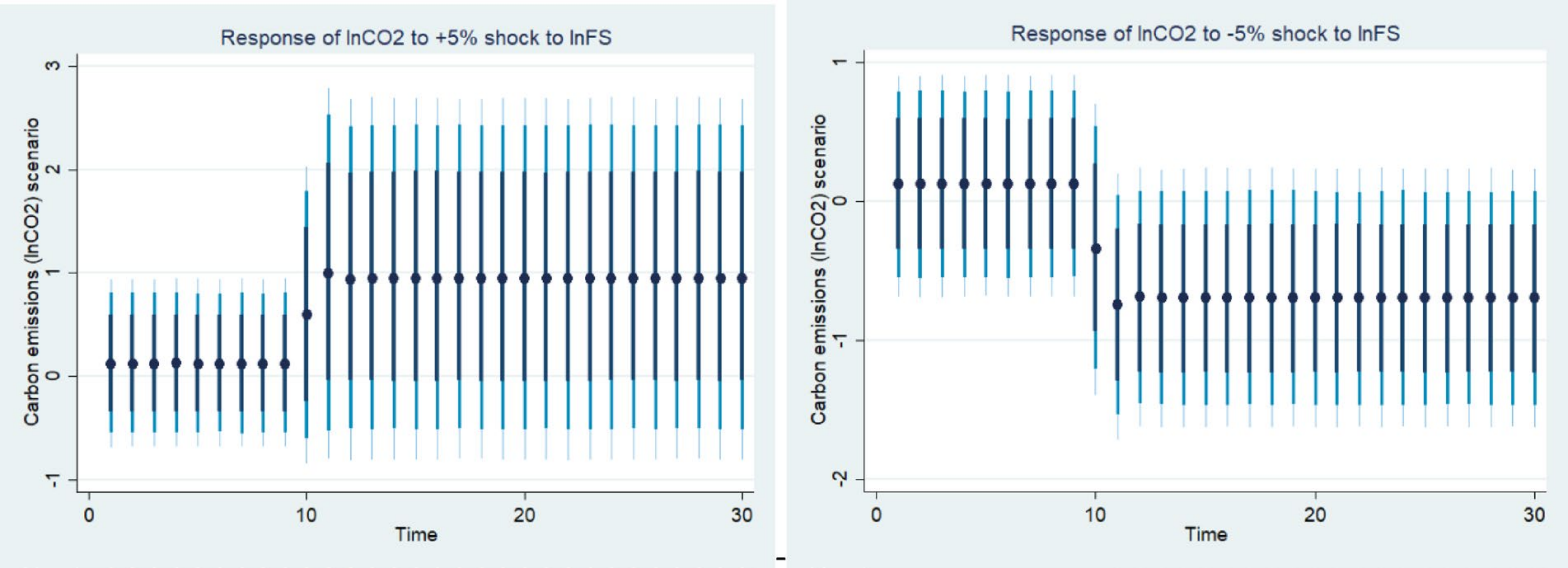
5% (±) shock to food security on carbon emissions yielded using the DARDL simulations area plot.

**Fig. 8:**
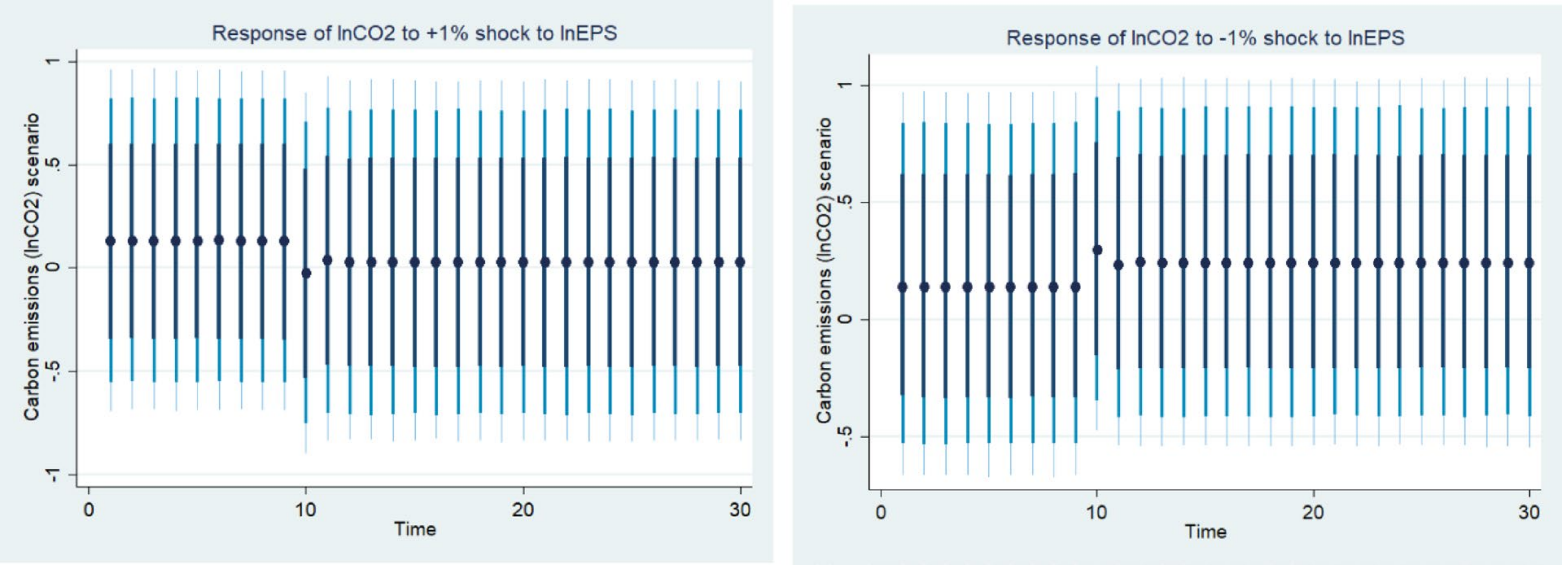
1% (±) shock to environmental policy stringency on carbon emissions yielded using the DARDL simulations area plot.

**Fig. 9:**
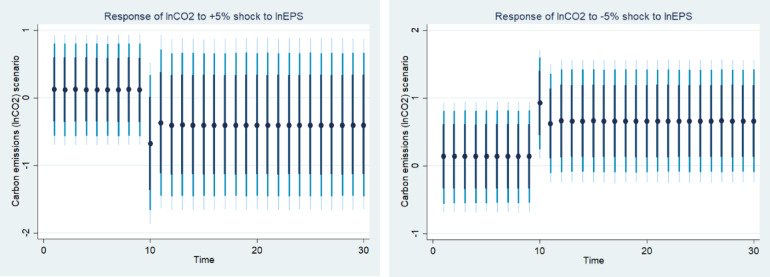
5% (±) shock to environmental policy stringency on carbon emissions yielded using the DARDL simulations area plot.

**Fig. 10:**
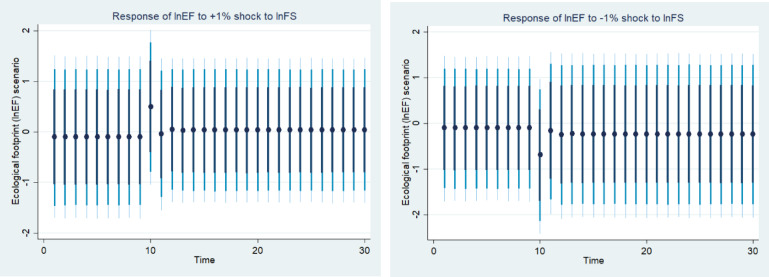
1% (±) shock to food security on ecological footprint yielded using the DARDL simulations area plot.

**Fig. 11:**
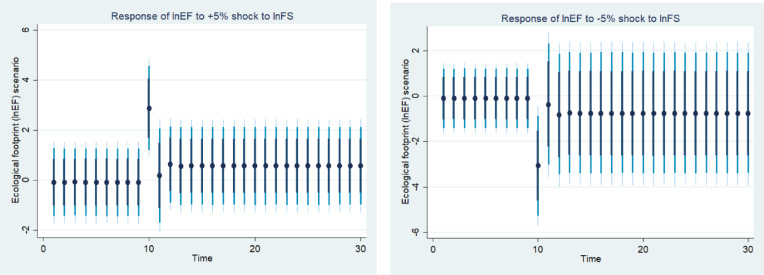
5% (±) shock to food security on ecological footprint yielded using the DARDL simulations area plot.

**Fig. 12:**
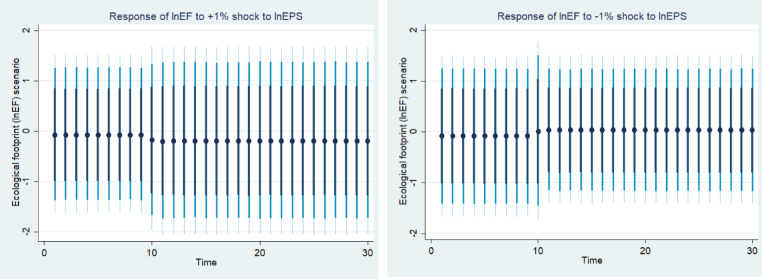
1% (±) shock to environmental policy stringency on ecological footprint yielded using the DARDL simulations area plot.

**Fig. 13:**
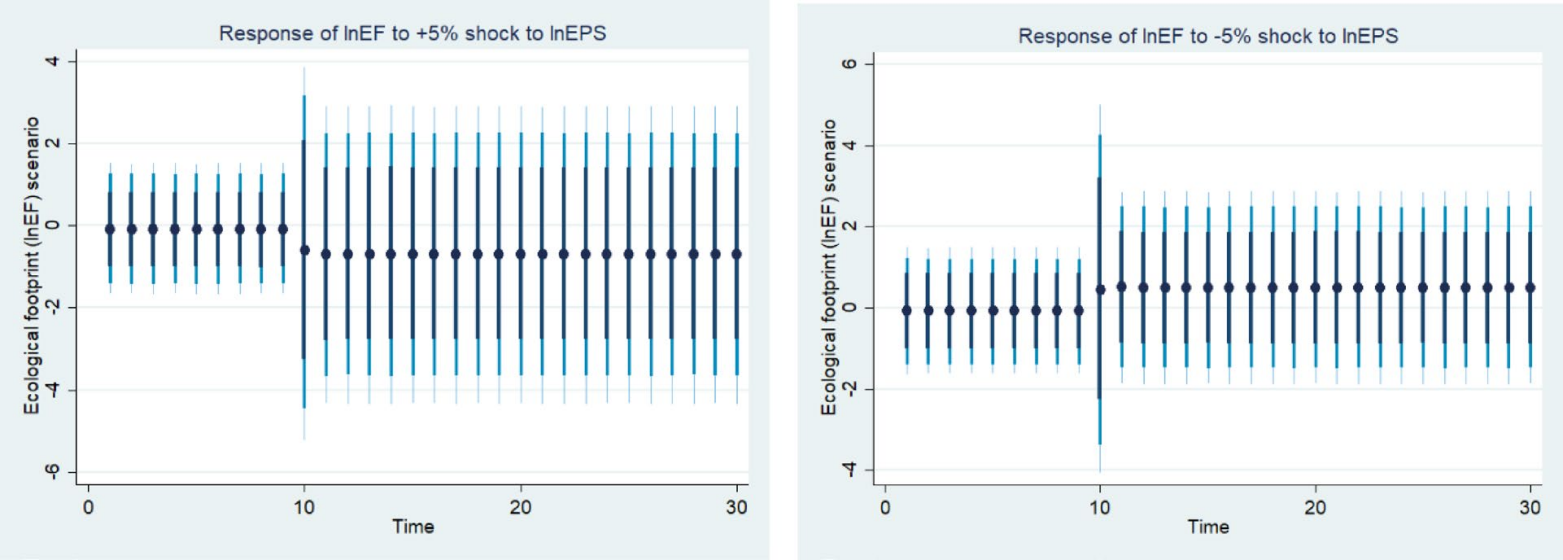
5% (±) shock to environmental policy stringency on ecological footprint yielded using the DARDL simulations area plot.

This study examines the responses of carbon emissions (*lnCO*_*2*_) and ecological footprint (*lnEF*) to variations in food security (*lnFS*) and environmental policy stringency (*lnEPS*) using a counterfactual simulation approach. The DARDL model generates plots depicting the projected impact of both positive and negative shocks to *lnFS* and *lnEPS* over a 30-year horizon. Figures 6, 7, 8, 9, 10, 11, 12, 13 display these simulation results, with the dots representing the mean predicted values and the shaded bands, ranging from deep blue to light blue, indicating the 75%, 90%, and 95% confidence intervals.

Figures 6 and 7 illustrate a gradual increase in *lnCO*_*2*_ following positive shocks of + 1% and + 5% to *lnFS*. In contrast, negative shocks of −1% and − 5% to *lnFS* lead to a consistent reduction in *lnCO*_*2*_. Over the long term, the impact of these shocks expands significantly as their magnitude grows from 1% to 5%.

Figures 8 and and 9 demonstrate a consistent decline in *lnCO*_*2*_ in response to positive shocks of + 1% and + 5% to *lnEPS*. In contrast, negative shocks of −1% and − 5% to *lnEPS* lead to a steady increase in *lnCO*_*2*_. Over the long term, the impact of these shocks becomes more pronounced as their magnitude intensifies from 1% to 5%. These findings are in line with the estimations derived from the DARDL simulation technique, underscoring the robustness of the results.

Figures 10, 11 illustrate a consistent rise in *lnEF* following positive shocks (+ 1% and + 5%) to *lnFS*. In contrast, negative shocks (−1% and − 5%) to *lnFS* lead to a steady decline in *lnEF*. Over the long term, the impact of these shocks becomes more pronounced as the magnitude increases from 1% to 5%.

Figures 12–13 demonstrate a steady decline in *lnEF* following positive shocks (+ 1% and + 5%) to *lnEPS*. Conversely, all negative shocks (−1% and − 5%) to *lnEPS* result in a consistent increase in *lnEF*. Over the long term, the magnitude of these shocks shows a notable expansion from 1% to 5%. These observations are consistent with the results derived from the DARDL simulation approach.

###  Machine learning approach with KRLS

This study investigates the influence of lnFS, lnEPS, lnEC, lnGPR, and lnTI on carbon emissions and ecological footprint using the KRLS machine learning algorithm. This approach utilizes pointwise differentials to analyze causal relationships among the variables. The results offer valuable insights at the 25th, 50th, and 75th percentiles, allowing for an assessment of the time-dependent marginal effects of the predictors on *lnCO*_*2*_ and *lnEF* through derivative analysis.

**Table 10:** KRLS-based pointwise derivatives.

Variable	Model 1						Model 2						
	Mean	SE	*P* > t	25th	50th	75th	Mean	SE	*P* > t	25th	50th	75th	
lnfs	0.087	0.023	0.001	0.189	0.136	0.029	0.009	0.004	0.021	0.020	0.006	0.000	
lneps	−0.214	0.047	0.000	−0.015	0.003	0.009	−0.034	0.013	0.019	−0.073	0.024	0.109	
lnec	0.154	0.029	0.000	0.018	0.201	0.254	0.270	0.064	0.000	−0.045	0.021	0.063	
lngpr	0.275	0.043	0.007	0.159	0.043	0.122	0.087	0.024	0.001	0.069	0.095	0.120	
lnti	−0.051	0.02	0.016	−0.02	−0.067	0.105	−0.372	0.162	0.030	−0.104	−0.045	0.039	
Diagnostics test													
$\:\lambda\:$	Tolerance	$\:\sigma\:$	Eff. Df.	R-sq	Looloss	N	$\:\lambda\:$	Tolerance	$\:\sigma\:$	Eff. Df.	R-sq	Looloss	N
0.097	0.031	5	15.7	0.932	0.367	33	0.301	0.031	4	11.14	0.865	0.465	33

The pointwise differentials from Models 1 and 2, presented in Table 10, highlight the impact of food security and environmental policy stringency on carbon emissions and ecological footprint in the context of geopolitical risks in the long run. Findings indicate that *lnFS*, *lnEC*, and *lnGPR* are positively and statistically significantly associated with *lnCO*_*2*_ and *lnEF*, whereas *lnEPS* and *lnTI* show a significant negative relationship. The diagnostic statistics indicate that both models are well-estimated and reliable, with identical tolerance levels confirming stable convergence. Model 1, characterized by a lower regularization parameter (λ) and higher effective degrees of freedom, exhibits greater flexibility, resulting in a superior goodness-of-fit (R² = 0.932) and lower leave-one-out loss, suggesting stronger predictive performance. Also, Model 2 applies stronger regularization, yielding a more parsimonious specification with reduced complexity, albeit with a modest decline in explanatory power (R² = 0.865) and predictive accuracy.

**Fig. 14:**
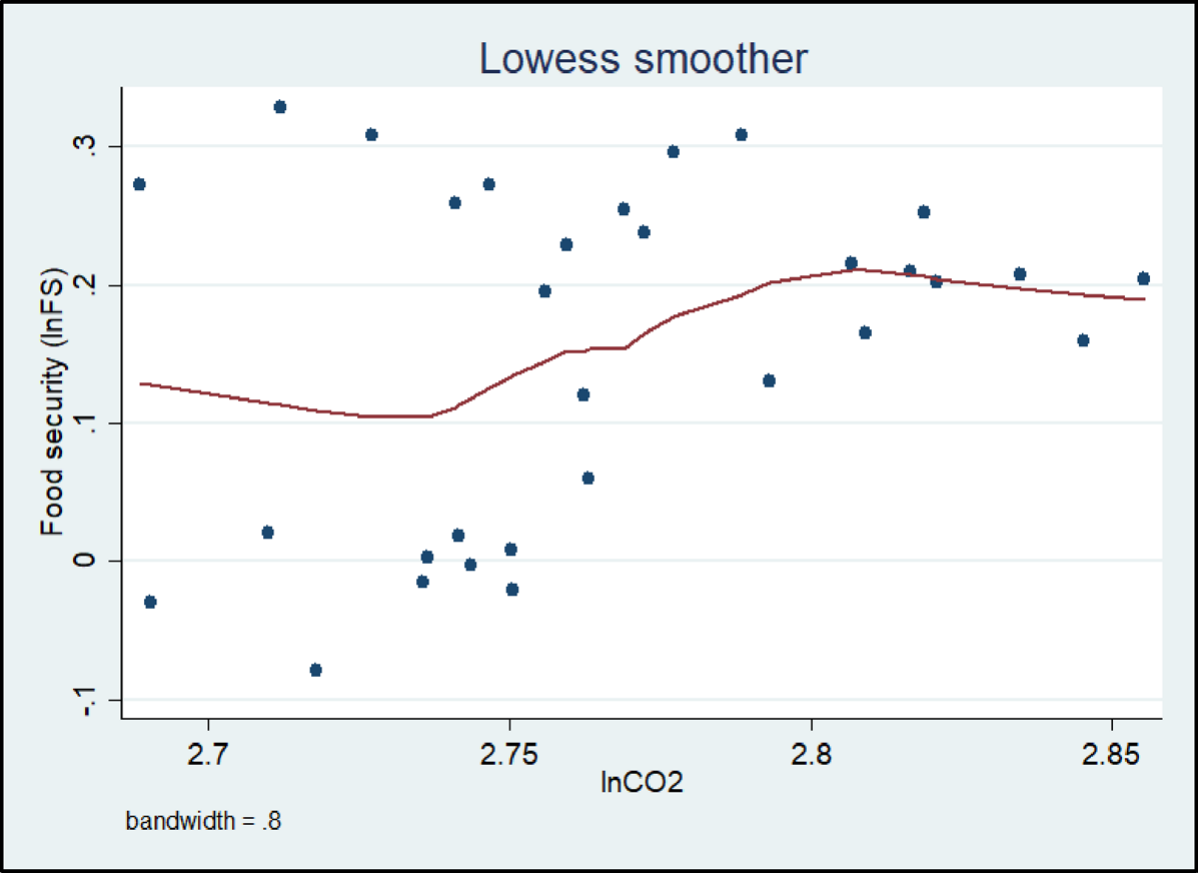
Pointwise marginal effect of food security (*lnFS*) on carbon emissions (*lnCO*_*2*_).

**Fig. 15:**
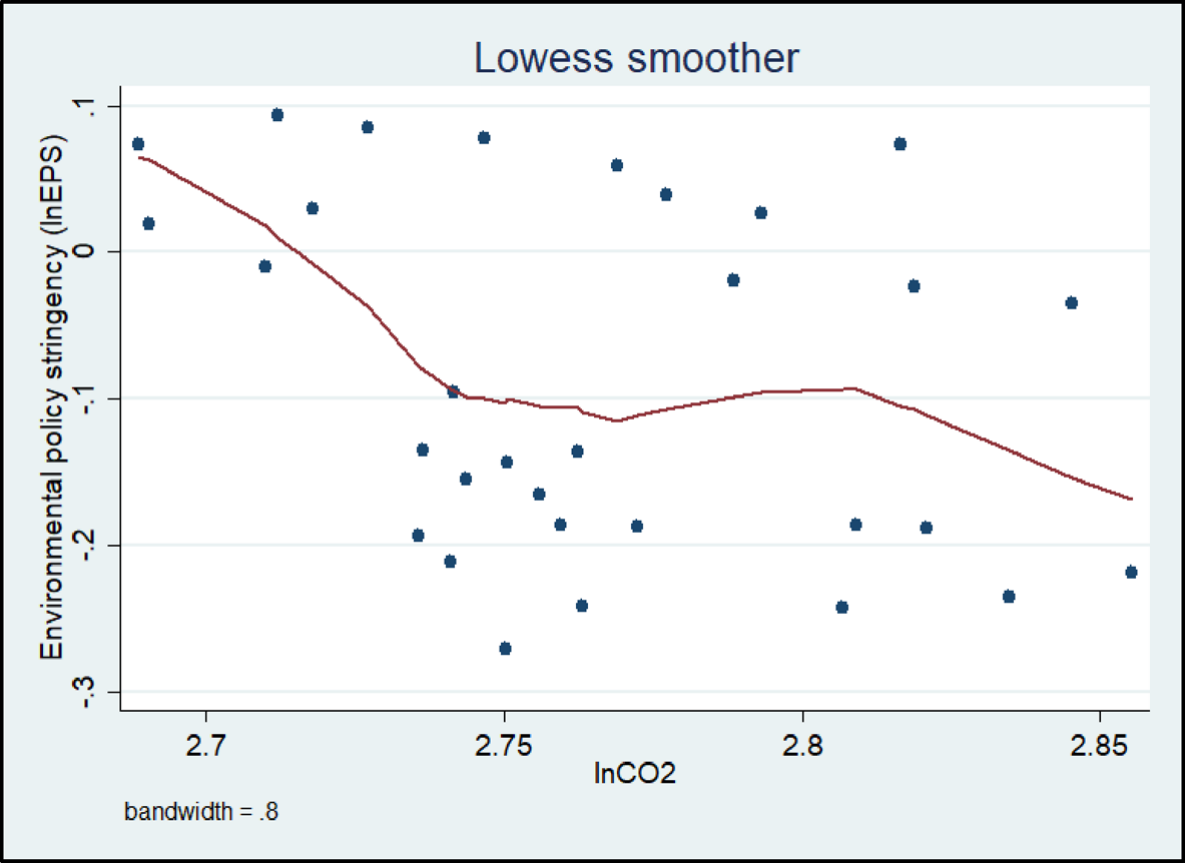
Pointwise marginal effect of environmental policy stringency (*lnEPS*) on carbon emissions (*lnCO*_*2*_).

**Fig. 16:**
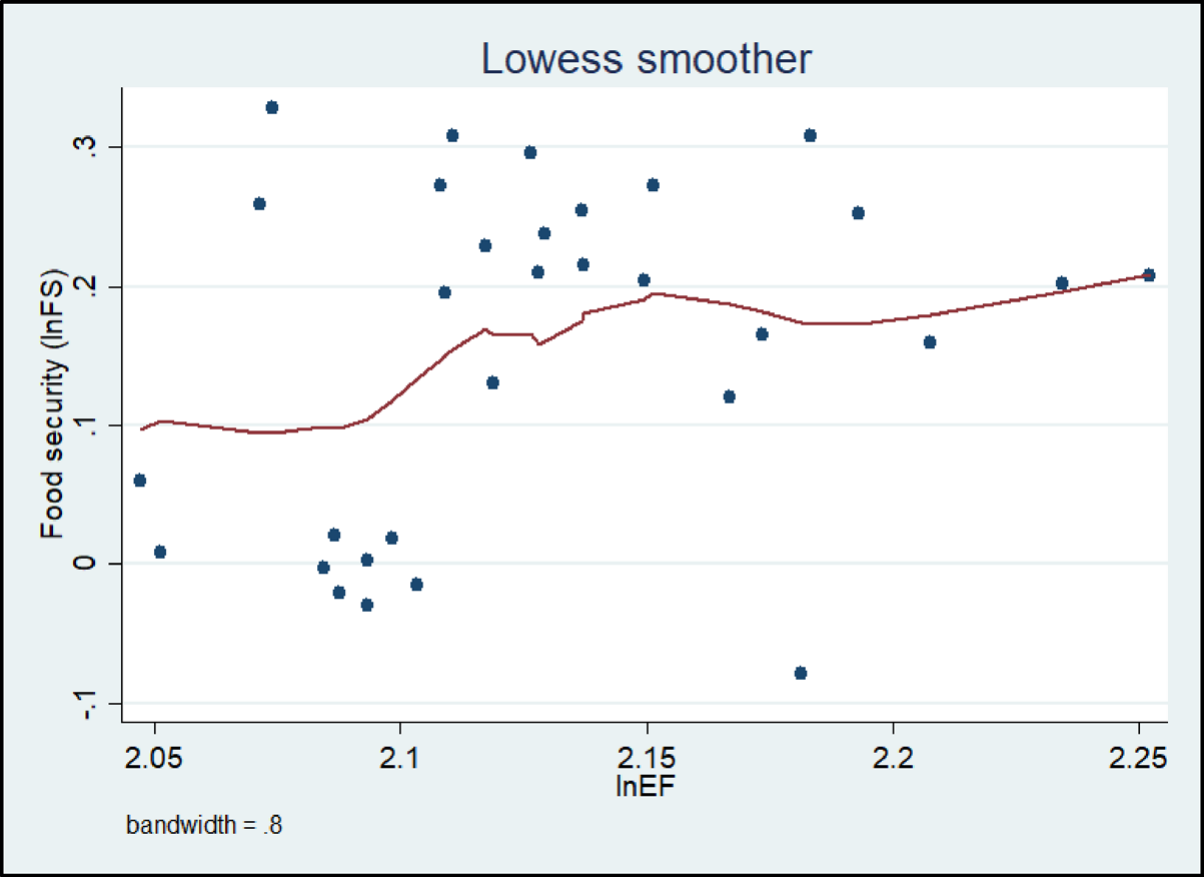
Pointwise marginal effect of food security (*lnFS*) on ecological footprint (lnEF).

**Fig. 17:**
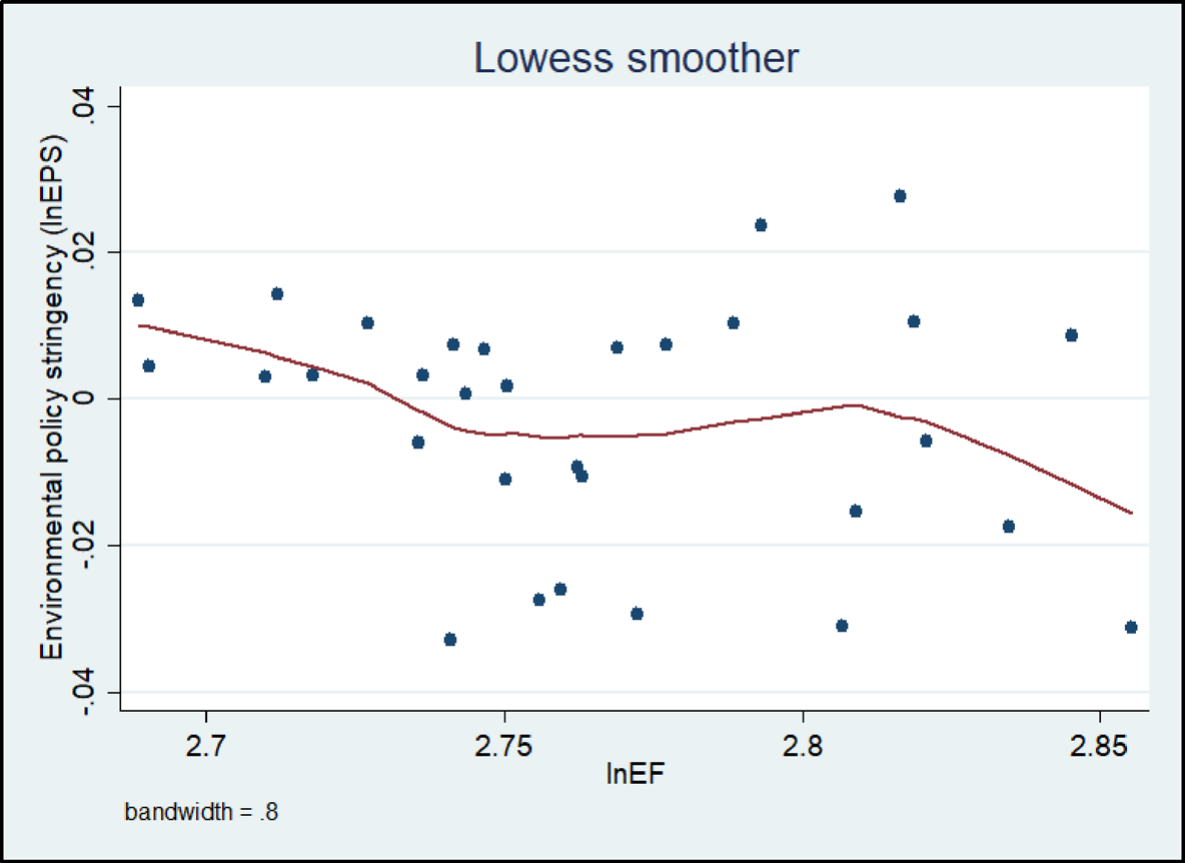
Pointwise marginal effect of environmental policy stringency (*lnEPS*) on ecological footprint (*lnEF*).

Next, this study explores the long-term changes in *lnFS* and *lnEPS* and their impact on *lnCO*_*2*_ and *lnEF* as illustrated in Figs. 14, 15, 16 and 17.

Figure 14 illustrates that the positive marginal effect of *lnFS* remains consistent as *lnCO2* levels rise, showing a gradual upward trend in *lnCO2* magnitude, with a slight decline toward the end. These results suggest that *lnFS* continues to have an increasingly significant marginal impact as carbon emissions grow, which is consistent with the findings from the DARDL analysis.

Figure 15 demonstrates a decreasing trend in environmental policy stringency as carbon emissions rise. The figure highlights a sharp decline in *lnEPS* at lower *lnCO*_*2*_ values, followed by a flatter trajectory and gradual decline at higher *lnCO*_*2*_ levels. This trend suggests that stricter environmental policies correlate with lower carbon emissions, supporting findings from the co-integration and counterfactual shock analyses conducted using the DARDL approach.

Figure 16 illustrates a positive a positive marginal relationship between food security and ecological footprint. The figure shows an initial flat trend followed by a steady increase in *lnFS* as *lnEF* rises, with minor fluctuations. This trend suggests that food security accelerates progressively with increasing ecological footprint, aligning with the findings from the DARDL analysis.

Figure 17 shows a decreasing trend in environmental policy stringency as the ecological footprint rises. The figure highlights an initial gradual decline in *lnEPS*, followed by a relatively stable phase and a further drop at higher *lnEF* levels. These findings suggest that stricter environmental policies are associated with lower ecological footprint levels, consistent with the co-integration and counterfactual shock analyses using the DARDL approach.

**Table 11:** Summary of Tests, Estimation Methods, and Key Findings.

Category	Method/Test	Key Results	Interpretation
Descriptive Analysis	Summary Statistics	Low variation in lnCO2; moderate variation in others; Jarque-Bera insignificant	Data is stable and normally distributed
	Correlation Matrix	lnCO2 positively correlated with FS, EC, GPR; negatively with EPS, TI	Initial evidence of environmental drivers and mitigators
Stationarity Test	ADF & PP Tests	Mixed integration: lnFS is I(0); others I(1)	Suitable for ARDL/DARDL modeling
Cointegration Test	PSS Bounds Test	Model 1 (F = 8.704***), Model 2 (F = 9.853***) → cointegration confirmed	Long-run equilibrium relationship exists
Estimation Method	DARDL Model	Significant long-run relationships; strong ECT (−0.813, −0.765)	Rapid adjustment toward equilibrium
Main Long-run Findings	DARDL Results	FS (+), EC (+), GPR (+); EPS (−), TI (−)	Food security and energy increase degradation; policy and innovation reduce it
Short-run Dynamics	DARDL Short-run	FS and GPR significant for CO2; FS and EPS for EF	Short-run effects are variable-specific
Diagnostics Tests	Autocorrelation (LM Test)	No autocorrelation (*p* > 0.05)	Model is reliable
	Heteroskedasticity (IM Test)	Homoscedastic residuals	No variance bias
	Normality Test	Residuals normally distributed	Valid inference
	Stability Test (CUSUM)	Stable within 95% bounds	Model structurally stable
Simulation Analysis	DARDL Counterfactual	FS shocks ↑ emissions; EPS shocks ↓ emissions	Confirms asymmetry and robustness
Machine Learning	KRLS	FS, EC, GPR (+); EPS, TI (−)	Confirms DARDL results
KRLS Diagnostics	Regularization & Fit	High *R*² (0.932, 0.865); stable parameters	Strong predictive accuracy

## Discussion

The empirical findings of this study provide important insights into the complex interactions between food security, environmental policy stringency, energy consumption, geopolitical risks, and technological innovation in shaping environmental quality in Canada.

First, the results indicate that food security significantly increases both carbon emissions and ecological footprint in the long run. This finding suggests that improvements in food security are associated with higher environmental pressure, reflecting the resource-intensive nature of modern food systems. In the Canadian context, achieving higher food security involves increased agricultural production, extensive transportation networks, and energy-intensive processing activities, all of which contribute to environmental degradation. This outcome supports the argument that food security and environmental sustainability may involve inherent trade-offs, particularly in developed economies where large-scale production dominates. The finding is consistent with Sun et al.^49^, Niu et al.^50^ and He^51^, who also report a positive association between food security and environmental degradation.

Second, environmental policy stringency is found to significantly reduce carbon emissions and ecological footprint, confirming its effectiveness as a policy tool for environmental protection. This result supports the Porter Hypothesis, which posits that well-designed environmental regulations can stimulate innovation and improve environmental performance. In Canada, policies such as carbon pricing, emissions reduction targets, and renewable energy mandates appear to have successfully encouraged cleaner production and reduced reliance on carbon-intensive activities. This finding aligns with Yirong^9^, Li et al.^52^ and Dai and Du^53^, highlighting the role of stringent environmental policies in improving environmental outcomes across different economic contexts.

Third, energy consumption emerges as the most dominant driver of environmental degradation. The strong positive impact of energy consumption on both carbon emissions and ecological footprint reflects Canada’s continued reliance on fossil fuel-based energy sources, particularly in industrial, transportation, and residential sectors. Despite progress in renewable energy adoption, the current energy mix remains insufficient to offset environmental damage. This finding is consistent with the energy–environment nexus literature, including Karaaslan and Camkaya^5^and Çamkaya et al.^4^,, as well as empirical evidence from Kirikkaleli et al.^54^, and Shah et al.^55^, which emphasize the environmental costs of energy-intensive growth.

Fourth, geopolitical risks are found to exacerbate environmental degradation, particularly through their substantial impact on ecological footprint. This suggests that geopolitical instability disrupts energy markets, trade flows, and resource allocation, leading to inefficient production and increased environmental pressure. In periods of geopolitical uncertainty, countries may prioritize energy security over environmental sustainability, resulting in greater reliance on domestic fossil fuel resources and delayed adoption of green technologies. This finding is in line with Ding et al.^56^,Li et al.^57^, and Villanthenkodath and Pal)^58^, who highlight the environmental implications of geopolitical tensions.

Fifth, technological innovation significantly improves environmental quality by reducing both carbon emissions and ecological footprint. This indicates that advancements in clean technologies, renewable energy systems, and energy-efficient processes play a crucial role in mitigating environmental degradation. In Canada, investments in innovation—particularly in green technologies—appear to support sustainable production and resource optimization. This finding is consistent with Dong et al.^59^, Saqib and Dincă^60^, and Nketiah et al.^61^, as well as recent evidence emphasizing the role of eco-friendly technologies in improving environmental outcomes^4,12^.

## Conclusions and policy recommendations

This study investigates the relationship between food security, environmental policy stringency, energy consumption, geopolitical risks, and technological innovation on environmental quality—proxied by carbon emissions and ecological footprint—within the Canadian context. Using annual time series data from 1990 to 2022, the study applies the dynamic autoregressive distributed lag approach to examine long-run and counterfactual relationships, complemented by the Kernel-based Regularized Least Squares method to ensure robustness. The findings reveal that food security, energy consumption, and geopolitical risks exacerbate environmental degradation, while environmental policy stringency and technological innovation significantly improve environmental quality. Diagnostic and stability tests further confirm the reliability and robustness of the estimated models.

The findings offer several important policy implications. First, the positive relationship between food security and environmental degradation suggests that achieving food security through conventional, resource-intensive methods may come at a significant environmental cost. Therefore, policymakers should prioritize the development of sustainable and resilient food systems, including the adoption of climate-smart agriculture, reduction of food waste, and promotion of localized food production. Encouraging innovations in agricultural technologies that improve productivity while minimizing resource use is critical. Second, the effectiveness of environmental policy stringency in reducing emissions and ecological footprint underscores the importance of strengthening regulatory frameworks. Governments should enhance carbon pricing mechanisms, enforce stricter emission standards, and promote renewable energy adoption. In addition, targeted incentives—such as subsidies and tax benefits—should be provided to encourage firms to adopt green technologies and sustainable production practices.

Third, given the dominant role of energy consumption in driving environmental degradation, a transition toward cleaner energy systems is essential. Policymakers should accelerate investments in renewable energy sources, improve energy efficiency across sectors, and reduce dependence on fossil fuels. Integrating energy policy with environmental objectives will be crucial for achieving long-term sustainability. Fourth, the findings highlight the environmental implications of geopolitical risks, suggesting the need to strengthen energy security and international cooperation. Diversifying energy sources, stabilizing supply chains, and engaging in multilateral environmental agreements can help mitigate the adverse effects of geopolitical uncertainty on environmental outcomes. Finally, technological innovation emerges as a key driver of environmental sustainability. Thus, governments should foster innovation ecosystems by investing in research and development, supporting green technologies, and facilitating knowledge transfer across sectors.

Despite its contributions, this study has several limitations that provide avenues for future research. First, the analysis is limited to a single country (Canada), which may restrict the generalizability of the findings to other economic and institutional contexts. Future studies should extend the analysis to cross-country or panel data frameworks, particularly across OECD or emerging economies, to enhance external validity. Second, while this study incorporates geopolitical risks, it does not explicitly disentangle the specific transmission channels—such as energy trade disruptions or supply chain shocks—through which these risks influence environmental outcomes. Future research could incorporate case studies or sectoral analyses to provide deeper mechanistic insights. Third, the study focuses on aggregate indicators and does not account for sector-specific dynamics, such as differences between agricultural, industrial, and energy sectors. Future research could explore disaggregated data to better understand sectoral contributions to environmental degradation.

This study addresses a critical gap in the literature by jointly examining food security, environmental policy stringency, and geopolitical risks within a unified empirical framework. It also contributes methodologically by applying the DARDL approach alongside machine learning techniques, which remain underutilized in environmental economics. By integrating economic, environmental, and geopolitical dimensions, the study provides a more comprehensive understanding of the trade-offs and synergies involved in achieving sustainable development. In conclusion, the findings emphasize that while food security remains a fundamental development goal, it must be pursued alongside strong environmental policies, technological innovation, and sustainable energy transitions to ensure long-term environmental sustainability.

## Figures and Tables

**Fig. 18:**
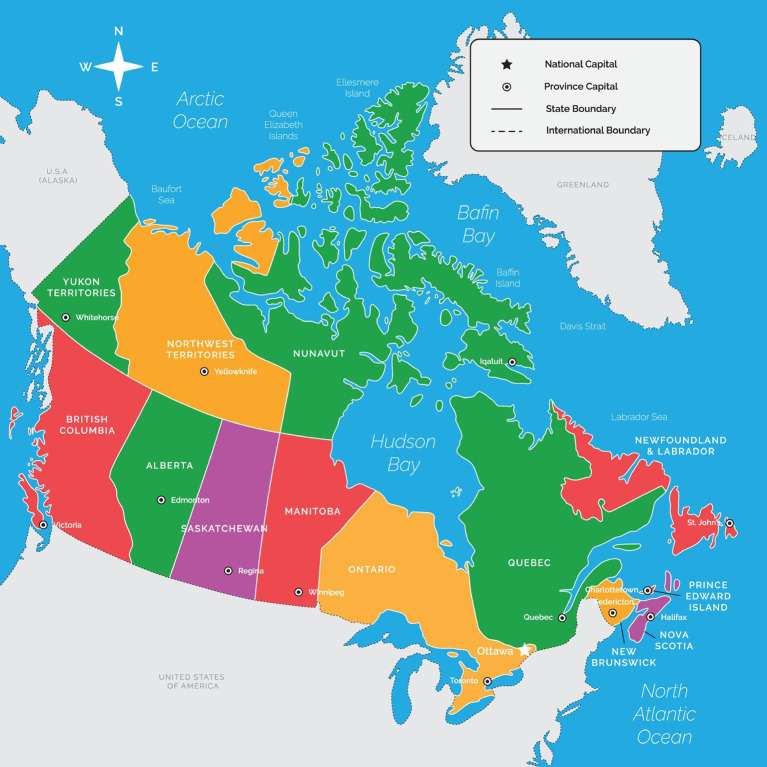
Map of Canada. (Source: www.vecteezy.com/vector-art/21523296-detailed-map-of-canada).

## Data Availability

Data used in this study are publicly available and can be accessed through the following websites:1. Carbon dioxide emissions: [https://data.worldbank.org/indicator/EN.GHG.CO2.PC.CE.AR5?locations=CA](https:/data.worldbank.org/indicator/EN.GHG.CO2.PC.CE.AR5?locations=CA)2. Ecological footprint: [https://data.footprintnetwork.org](https:/data.footprintnetwork.org)3. Food security: [https://data.worldbank.org/indicator/AG.PRD.FOOD.XD? locations=CA](https:/data.worldbank.org/indicator/AG.PRD.FOOD.XD? locations=CA)4. Environmental policy stringency: [https://data-explorer.oecd.org/vis? tm=environmental%20policy%20stringency%20index&pg=0&snb=1&df[ds]=dsDisseminateFinalDMZ&df[id]=DSD\_EPS%40DF\_EPS&df[ag]=OECD.ECO.MAD&df[vs]=1.0&dq=.A.EPS&lom=LASTNPERIODS&lo=5&to[TIME\_PERIOD]=false&vw=tb](https:/data-explorer.oecd.org/vis?tm=environmental%20policy%20stringency%20index&pg=0&snb=1&df%5bds%5d=dsDisseminateFinalDMZ&df%5bid%5d=DSD_EPS%40DF_EPS&df%5bag%5d=OECD.ECO.MAD&df%5bvs%5d=1.0&dq=.A.EPS&lom=LASTNPERIODS&lo=5&to%5bTIME_PERIOD%5d=false&vw=tb)5. Total energy consumption: [https://www.eia.gov/international/overview/country/CAN](https:/www.eia.gov/international/overview/country/CAN)6. Global geopolitical risk index: [https://www.matteoiacoviello.com/gpr.htm](https:/www.matteoiacoviello.com/gpr.htm)7. Technology innovation: [https://data.worldbank.org/indicator/IP.PAT.RESD](https:/www.data.worldbank.org/indicator/IP.PAT.RESD).

## Appendix

See Fig 18
